# Orientation-distribution mapping of polycrystalline materials by Raman microspectroscopy

**DOI:** 10.1038/srep18410

**Published:** 2015-12-17

**Authors:** T. Schmid, N. Schäfer, S. Levcenko, T. Rissom, D. Abou-Ras

**Affiliations:** 1 Federal Institute for Materials Research and Testing, Richard-Willstätter-Str. 11, 12489 Berlin, Germany; 2 Helmholtz-Zentrum Berlin für Materialien und Energie GmbH, Hahn-Meitner-Platz 1, 14055 Berlin

## Abstract

Raman microspectroscopy provides the means to obtain local orientations on polycrystalline materials at the submicrometer level. The present work demonstrates how orientation-distribution maps composed of Raman intensity distributions can be acquired on large areas of several hundreds of square micrometers. A polycrystalline CuInSe_2_ thin film was used as a model system. The orientation distributions are evidenced by corresponding measurements using electron backscatter diffraction (EBSD) on the same identical specimen positions. The quantitative, local orientation information obtained by means of EBSD was used to calculate the theoretical Raman intensities for specific grain orientations, which agree well with the experimental values. The presented approach establishes new horizons for Raman microspectroscopy as a tool for quantitative, microstructural analysis at submicrometer resolution.

Raman spectroscopy is a standard tool for materials characterization of inorganic as well as organic solids. This technique is based on Raman scattering of incident light by phonons and can provide information on local changes of composition, strain, and impurities via evaluation of corresponding lattice vibrations^1^.

It is known since the 1930s (e.g., ref. 2) that the Raman scattering intensity from a crystal is influenced by the orientation of the crystal with respect to the directions of light incidence and signal observation. This influence is due to the fact that Raman scattering is based on the coupling of the electrical field of the incident light with the electric moments of the scattering medium^1^. Consequently, the Raman intensity is also dependent on the direction of the polarization of the incident and of the scattered light. The Raman effect in crystals was reviewed by, e.g., Loudon^3^.

The combination of Raman spectroscopy with an optical microprobe setup has allowed for determining local crystal orientations nondestructively on micrometer-sized specimen areas^4^,^5^,^6^,^7^. This approach has been used to acquire Raman intensity-distribution maps on polycrystalline material systems^8^,^9^,^10^,^11^,^12^,^13^,^14^. Further work comprised Raman mapping with complementary electron microscopy analysis^15^. However, up to now, there has not been any effort to evaluate Raman maps on polycrystalline material systems quantitatively, and it has been possible to identify only a few grains within the reported Raman intensity-distribution maps^10^,^11^,^12^,^14^. Still, the presence of grains has not been verified at the same identical positions by means of an independent analytical method, such as electron backscatter diffraction (EBSD).

The present work raises Raman microspectroscopy to a new level as tool for the acquisition of orientation distributions of polycrystalline materials, showing that contiguous microstructure overviews on areas of several hundreds of μm^2^ at the submicrometer level can be obtained, containing quantitative information on the local orientations of individual grains. As a model system, a CuInSe_2_ thin film, applied as absorber material for solar cells, with an average grain size of about 1 μm, was used. CuInSe_2_ crystallizes in a tetragonal, chalcopyrite-type crystal structure with a lattice-constant ratio *c*/*a* very close to 2 (deviation of only about 0.2% (ref. 16)).

## Results and Discussion

### Raman spectra

**Figure 1:**
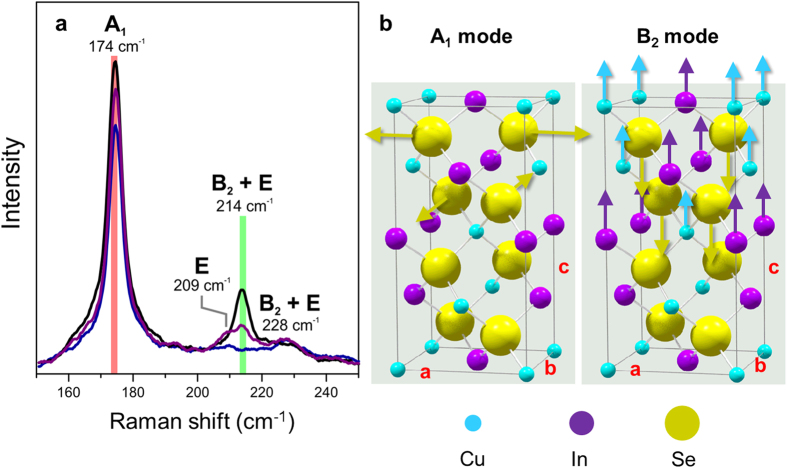
Exemplary Raman spectra from CuInSe_2_ crystals, with two major peaks at 174 and at 214 cm^−1^, as well as the representations of the corresponding atomic lattice vibrations. (**a**) Three Raman spectra from CuInSe_2_ crystals (represented in black, purple, and blue colors), acquired at different specimen positions. Two major peaks at 174 (A_1_ mode) and at 214 cm^−1^ (B_2_/E modes) as well as minor peaks at 209 (E modes) and 228 cm^−1^ (B_2_/E modes) were detected. (**b**) Representations of lattice vibrations in the tetragonal CuInSe_2_ unit cell, with lattice parameters *a*, *b*( = a), and *c*, giving the vibrations corresponding to the A_1_ and the B_2_ modes.

Exemplary Raman spectra, acquired at various positions on a polycrystalline CuInSe_2_ thin film, are given in Fig. 1a. They exhibit two major peaks at 174 and 214 cm^−1^, as well as minor peaks at 209 and 228 cm^−1^, corresponding to the A_1_ and B_2_/E vibrational modes^17^. The related lattice vibrations are represented in Fig. 1b. Since the expected peak at 209 cm^−1^ related to a purely E vibrational mode is rather small as compared with the one at 214 cm^−1^ (B_2_/E modes) in Fig. 1a, we assume that the Raman intensity for the B_2_ mode is much larger than that for the E mode. In the following, we will therefore concentrate only on contributions by A_1_ and B_2_ modes.

### Raman composite and EBSD orientation-distribution maps

**Figure 2:**
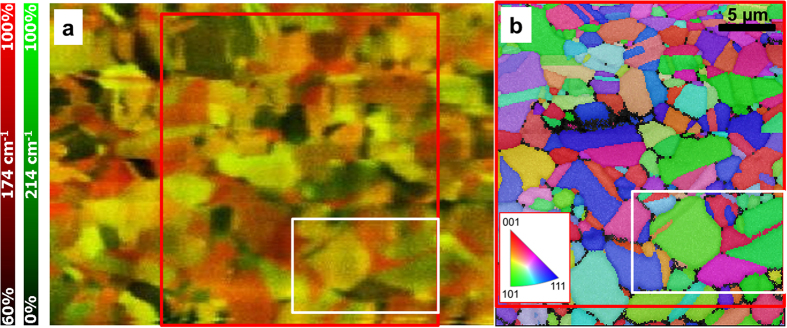
Overview Raman intensity and EBSD orientation-distribution maps, acquired on a polycrystalline CuInSe_2_ thin film. (**a**) Composite Raman intensity-distribution map using the signals at 174 (red) and at 214 cm^−1^ (green). (**b**) EBSD orientation-distribution map from the same identical specimen position as in (**a**). The local orientations are given in false colors (see legend); note that owing to the lattice-constant ratio *c*/*a* very close to 2, a cubic lattice was assumed for the (actually) tetragonal CuInSe_2_ crystal. In both, (**a**) and (**b**), a large, red and a small white frame highlight identical regions.

In order to obtain a contiguous map, Raman spectra were acquired at 200 × 200 individual measurement points (632.8 nm laser wavelength, 1 mW laser power, 100  × /N.A. = 0.9 objective lens, 5 s acquisition time per spectrum). The composite Raman map in Fig. 2a consists of the superimposed intensity distributions of the Raman signals recorded at 174 and at 214 cm^−1^. From its intensity distribution, this map gives rise to the assumption that it represents the spatial distribution of individual grains on the measured area. In order to verify this assumption, an EBSD orientation distribution map was acquired on the same identical position (Fig. 2b). Indeed, within the area highlighted by red frames in Fig. 2a,b, the shapes of the individual grains are similar in both, the Raman and the EBSD maps. Note that for the representation of the EBSD data, a quasicubic crystal structure was used for simplicity. The microstructure of the polycrystalline CuInSe_2_ thin film is reproduced well even for small grains with diameters well below 1 μm.

From the analysis of small crystallographic features (twin lamellae) in the Raman map, lateral resolutions of about 400 nm were determined (Fig. S2). Also, by repeated acquisitions of Raman maps on the same identical specimen positions, high reproducibility was demonstrated (Fig. S5). Moreover, from the Raman intensity distributions, also strain distributions within individual grains were extracted, with spectral resolutions of about 0.3 cm^−1^ per pixel (on the charge-coupled device camera), which is equivalent to a strain value in the order of 10^-3^. Further details can be found the Supplementary Materials of the present contribution.

### Raman intensity distributions for various polarization directions and wavelengths

**Figure 3:**
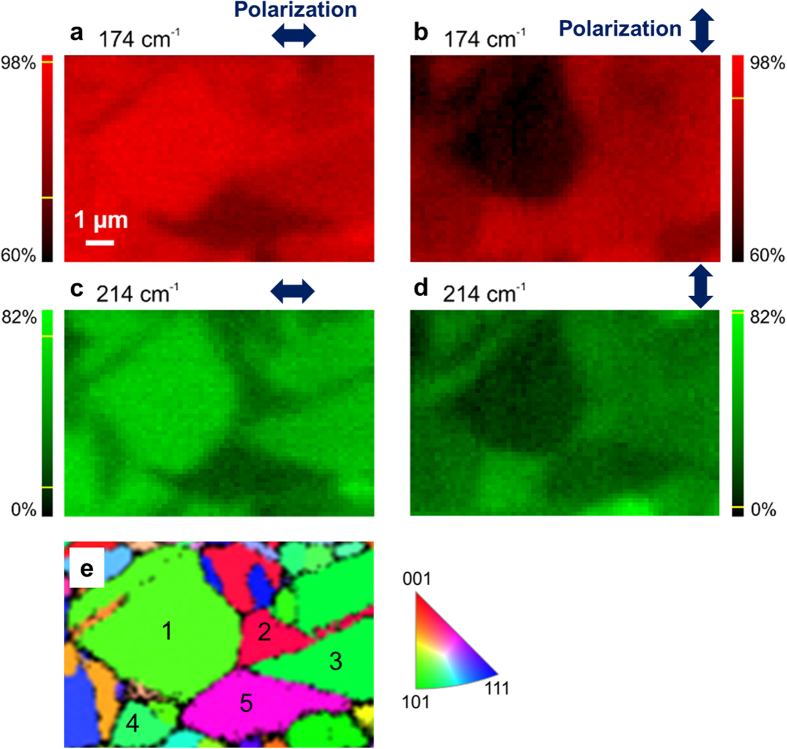
Raman intensity and local orientation distribution maps from the same identical position, as well as orientations of the grains 1–5. Normalized Raman intensity distributions at 174 (**a**,**b**) as well as at 214 cm^−1^ (**c**,**d**) for two different orientations of the polarization of the incident laser light (**a**/**c**,**b**/**d**), acquired on the region highlighted in Fig. 2 by a white frame. The EBSD orientation distribution map from this region is given in (**e**), indicating five grains (1–5).

In the following, we will concentrate on the area highlighted by a white frame in Fig. 2. Raman intensity distribution maps were also acquired at various polarization directions of the incident laser light. Intensity-distribution maps using Raman signals at 174 and at 214 cm^−1^ as well as two polarization directions perpendicular to each other are shown in Fig. 3a–d. Substantial differences in Raman intensity for various grains at these two polarization directions were detected. We selected five grains, grain 1 to grain 5 in the EBSD orientation distribution map (Fig. 3e), in order to investigate this change in Raman intensity at different polarization directions more in depth.

### Comparison of experimental and theoretical Raman intensities

Although the shapes of the grains resemble each other well in the Raman and EBSD maps, the measured Raman intensities at 174 and at 214 cm^−1^ in the Raman map should also agree for various polarization directions with the theoretical intensities of the scattered light, which can be calculated by using the approach reported by Loudon^3^. Also Tanino *et al.*^18^ showed that the crystal orientations of CuInSe_2_ single crystals (in contrast to the polycrystalline thin film investigated in the present work) with respect to the incidence and scattering directions of the laser light can be calculated from Raman intensity, by recording Raman spectra on the single crystals at varying polarization directions and evaluating the Raman peaks corresponding to the various vibrational modes.

**Figure 4:**
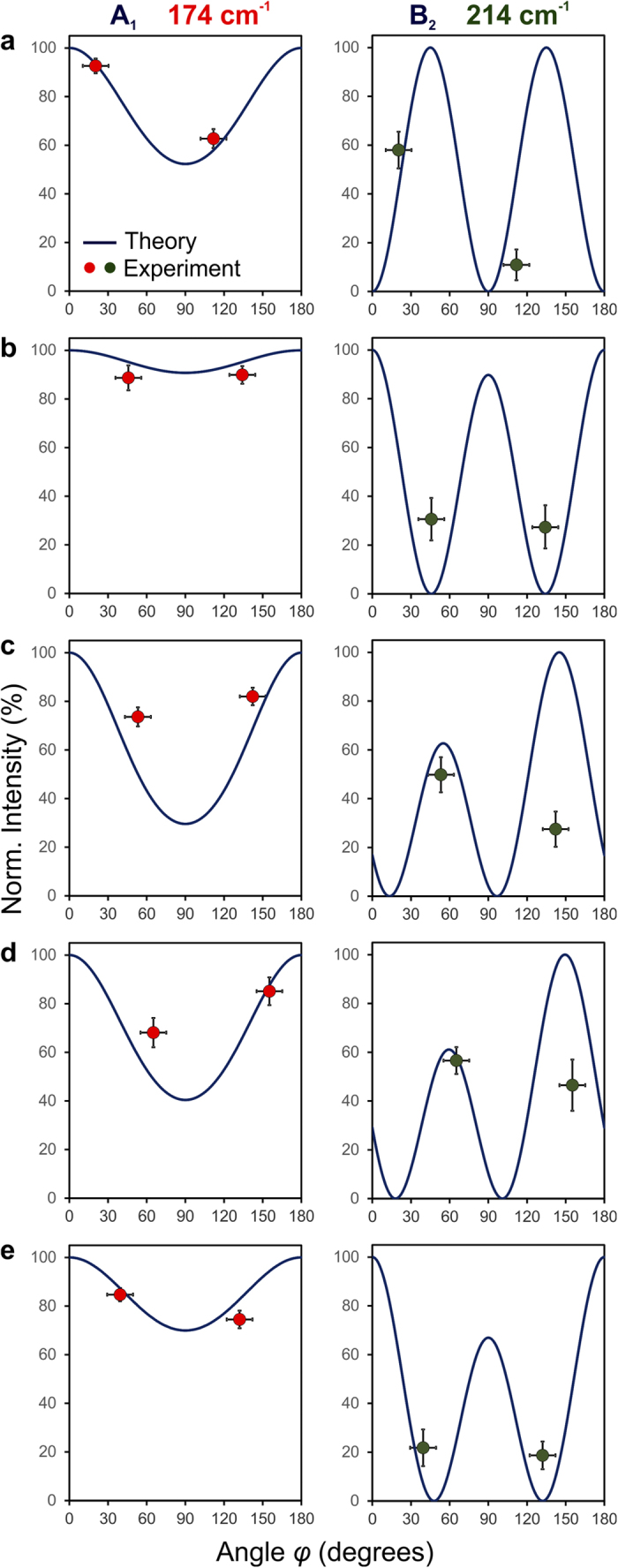
Theoretical Raman intensities, normalized to the maximum value, for the two polarization directions indicated in Fig. 3a–d, divided into contributions from the Raman signals at 174 and at 214 cm^−1^. The Raman intensities differ depending on the orientation of the CuInSe_2_ crystal lattice. The measured Raman intensities for the crystal orientations of grains 1–5 (**a–e**) are represented by red (174 cm^−1^) and green circles (214 cm^−1^).

Figure 4a–e give the theoretical Raman intensities for grains 1–5 indicated in Fig. 3a–d, divided into contributions from the Raman signals at 174 and at 214 cm^−1^. The Raman intensities differ depending on the orientation of the CuInSe_2_ crystal lattice. The measured Raman intensities for the crystal orientations of grains 1–5 are represented by red (174 cm^−1^) and green circles (214 cm^−1^). Especially for the most intensive Raman peak at 174 cm^−1^, a good agreement of experiment and simulation is found. The slight deviations visible for the 214 cm^−1^ peak can be explained by the considerably lower Raman intensities at this wavenumber, and also by the fact that the peak at 214 cm^−1^ contains contributions from the B_2_ and E modes, whereas only the B_2_-mode contribution was considered for the theoretical Raman intensities. (As supplementary information to the reader, the theoretical Raman intensities considering only the E mode as well as a linear combination with equally weighted contributions from B_2_ and E modes are given in Fig. S7.)

It should be noted that the presented approach to obtain orientation-distribution maps from polycrystalline materials by Raman microspectroscopy can by no means replace EBSD as standard tool for this purpose. This is true since optical diffraction limits the spatial resolution of Raman microspectroscopy to few hundreds of nanometers, while the spatial resolution for EBSD maps is at least one order of magnitude lower, and also since the material system under investigation needs to be Raman active. However, Raman intensity distribution maps containing superimposed contributions from various vibrational modes can provide, if measured on polycrystalline, multicomponent materials, not only orientation distributions, but also indicate (similar to EBSD maps) the presence and positions of secondary phases (e.g., ref. 14). Moreover, Raman imaging is closely related to conventional light microscopy, basically needs not any extensive sample preparation (such as polishing or chemical treatments), and can be performed at ambient conditions. When using tip-enhanced Raman spectroscopy (TERS), highest spatial resolutions of approximately 10 nm have already been achieved^19^. Corresponding improvements to the orientation-distribution analysis outlined in the present work can be expected when applying TERS to polycrystalline materials systems.

## Conclusions

Orientation-distribution maps were acquired on polycrystalline CuInSe_2_ thin films by means of Raman microspectroscopy. The corresponding Raman intensities on individual CuInSe_2_ grains agree well with calculated values using the local orientations determined by EBSD. The approach described in the present work can be applied to various polycrystalline material systems, as long as they are Raman active and the average grain sizes are appropriate.

## Materials and Methods

### Fabrication of CuInSe_2_/Mo/glass stacks

Samples were originally prepared as complete solar cells. Soda lime glass was employed as substrate material. The glass was cleaned using an alkaline and an acidic soak. Molybdenum was DC sputtered in a two-layer process. The CuInSe_2_ layer was prepared by co-evaporation of the elemental constituents from point sources in a vacuum system with a base pressure of about 1 × 10^−7^ mbar. While the In, Ga and Cu cells were open cells, the Se cell was a Knudsen type cell. Process control was achieved by laser light scattering measurements^20^. The process was similar to the NREL three-stage process^21^. Se was evaporated throughout the whole process duration. During the first stage the temperature of the sample heater was set to 330 °C (actual sample temperature might have been slightly lower) and In and Se were evaporated for 3333 s. The amount of deposited material could be estimated from the observed interference fringes. During the second stage with an increased substrate (heater-) temperature of 530 °C Cu and Se were deposited within 1169 s. A holding period of 300 s followed, in which only Se was evaporated. Afterwards In and Se were deposited for 893 s. Subsequently, only selenium was evaporated within 60 s with constant substrate temperature. During the following 1200 s the selenium flux was maintained constant while the substrate temperature was lowered to 200 °C.

The CuInSe_2_ layer was covered with a 50 nm thick CdS buffer layer, which was deposited in a chemical bath. A window layer consisting of 125 nm i-ZnO combined with 250 nm n-ZnO was applied through a RF sputtering process. Afterwards a grid consisting of 100 nm Ni and 2 μm of Al was deposited through a shadow mask by electron beam evaporation. The completed cells exhibited conversion efficiencies ranging from 12.2 to 13.7%.

The final composition was determined by X-ray fluorescence analysis on equivalent samples from the same process to [Cu] = 22%, [In] = 28%, and [Se] = 50%, which corresponds to a [Cu]/[In] ratio of 0.79.

### Sample preparation

For the conduction of the Raman and EBSD mapping, the ZnO and CdS layers were removed by etching using low-concentrated HCl and bromide reducing surface roughness. After etching, the surface quality of the samples was improved by a final polishing step using a colloidal silica suspension (OPS by Struers). A nominally 5 nm thick carbon layer was deposited on the examined surface to prevent charging commonly obtained at EBSD measurements.

### Conduction of Raman microspectroscopy

Raman spectra and maps presented here were collected using a LabRam HR 800 instrument (Horiba Jobin Yvon, Bensheim, Germany) coupled to a BX41 microscope (Olympus, Hamburg, Germany). A HeNe laser having a wavelength of 632.8 nm and a power of approx. 10 mW at the sample was used for excitation. The CuInSe_2_ samples were investigated using a 100x/N.A. = 0.9 objective for both, excitation and collection. Spectra were acquired by dispersing the collected light with a grating having 1800 grooves mm^−1^ (optionally, this can be replaced by a 300-mm^−1^ grating) and using a 1024 × 256-pixel CCD detector (Symphony, Horiba Jobin Yvon, liquid-N_2_ cooled, −126 °C operating temperature). With this configuration the spectral resolution is approx. 0.3 cm^−1^/CCD pixel (or approx. 2.5 cm^−1^/CCD pixel with the 300-mm^−1^ grating) at the position of the most prominent (A_1_) mode of the CuInSe_2_ spectrum. The laser power was attenuated to 1 mW by employing a neutral density filter, because sample damage was observed for laser powers exceeding 4 mW (Fig. S1). Raman maps were obtained by step-wise movement of the sample through the laser focus by a sample-scanning stage and collection of the whole Raman spectrum at every spot within typically 3 to 5 s (controlled by the instrument’s LabSpec software). Typical step sizes (corresponding to pixel sizes of the resulting Raman maps) ranged from 200 to 250 nm in both lateral directions.

### Raman microspectroscopy data analysis

Raman mapping experiments resulted in 3D data matrices containing a Raman spectrum in each pixel of a two-dimensional sample area. The matrices were converted to 2D images by using own (T.S., BAM, Berlin, Germany) LabVIEW-based (National Instruments Corp., Austin, Texas) software. Data processing includes smoothing of each spectrum by applying a moving average of ±4 CCD pixels. The 2D Raman images show the distributions of individual band intensities integrated over ±1 cm^−1^. Further to smoothing of the resulting Raman map, integration compensates for small shifts of the wavenumber positions of the bands, which appear due to local strain. Spectra with spikes due to cosmic rays, which interfered with the evaluated integration ranges, were excluded from data evaluation. The spectra (or corresponding pixels of the Raman maps, respectively) were deleted. In a further step of the data evaluation procedure, the average intensity of the eight surrounding pixels was assigned to each deleted pixel.

In the raw data, intensity values (i.e. the color scales of Raman maps) are expressed in arbitrary units (i.e. detector counts), which are specific to our instrument configuration. In order to convert them into more generally comparable values, which can be related to the theoretical trends as well (Fig. 4), each intensity value was normalized to the maximum detected intensity of the corresponding Raman band (174 cm^−1^ or 214 cm^−1^). Therefore, as a first step the minimum intensity in the 200 × 200-pixel map shown in Fig. 2 of 25 counts – corresponding to the background noise level – was subtracted from each signal intensity in all Raman maps presented in this study. The second step consisted of the normalization of the 174 cm^−1^ intensities to the maximum intensity of this band of 399 counts found in the 200 × 200-pixel map and of the 214 cm^−1^ intensities to the corresponding maximum value of 66 counts. Thus, each signal intensity – expressed in % – is related to the highest detected intensity of the corresponding band.

For comparison with the theoretical intensity values of these grains, experimental values of grain 1 and 2 (Fig. 4) were extracted from the maps shown in Fig. 3 by calculating the average of 6 × 6 pixels located in a square area in the center of each grain, well separated from the grain boundaries by several pixels, in order to avoid interferences from surrounding grains. The error bars along the intensity axis in Fig. 4 reveal three times the standard deviations of the 36 pixel values of each grain.

### Conduction of EBSD measurement

The EBSD analysis was carried out using a Zeiss Ultraplus scanning electron microscope equipped with an Oxford Instruments NordlysNano EBSD detector and Aztec as acquisition software. The measurement was performed at 20 kV acceleration voltage, at a probe current of about 50 nA and under 70° sample tilt. The map was acquired using a step size of 100 nm. With a square grid of 250 × 250 scanned points, a total area of 25 μm × 25 μm was mapped. The hardware binning of the EBSD detector was set to 4 × 4 to reduce the measurement duration and to avoid charging effects. Systematical misindexing, based on the pseudosymmetry of CuInSe_2_ (ref. 16), was refrained by considering a quasicubic crystal structure. Noise and spikes of the EBSD data were reduced using HKL Channel5 post acquisition software. The position of the previous Raman measurement was marked with 4 spots by the HeNe laser at full power of 10 mW leading to visible morphological changes within 10 s laser irradiation (Fig. S1c). Based on the measured orientations of the grains and the corresponding Euler angles, the required directions and planes were extracted for the two exemplary grains.

## Additional Information

**How to cite this article**: Schmid, T. *et al.* Orientation-distribution mapping of polycrystalline materials by Raman microspectroscopy. *Sci. Rep.* **5**, 18410; doi: 10.1038/srep18410 (2015).

## Supplementary Material

Supplementary Information
