# Supplementary material for: Orientation-distribution mapping of polycrystalline materials by Raman microspectroscopy
Source: Sci Rep. 2015 Dec 17;5:18410. doi: 10.1038/srep18410 (PMC4682063; doi:10.1038/srep18410)
Supplement: Supplementary Information [file srep18410-s1.pdf]

Supplementary Information for

Orientation-distribution mapping of polycrystalline materials by Raman microspectroscopy

T. Schmid[1], N. Schäfer[2], S. Levcenko[2], T. Rissom[2], D. Abou-Ras[2,*]

[1]BAM Federal Institute for Materials Research and Testing, Richard-Willstätter-Str. 11, 12489 Berlin, Germany

[2]Helmholtz-Zentrum Berlin für Materialien und Energie GmbH, Hahn-Meitner-Platz 1, 14055 Berlin, Germany

*corresponding author: daniel.abou-ras@helmholtz-berlin.de

Lateral resolution of Raman microspectroscopy

The lateral resolution of an optical microscopy experiment can be calculated based on the Rayleigh criterion[22]:

$$d_{resol} = 0.61\frac{\lambda}{N.A.}, \quad (1)$$

where d_{resol} is the smallest distance of two microscopic objects, which are considered just resolved (intensity maximum of one object coinciding with the first minimum of the Airy disk of the other), λ is the wavelength of light, and *N.A.* is the numerical aperture of the objective lens. According to the Abbe criterion[23], the radius of a focus spot (base width/2) is

$$r_{focus} = 0.5\frac{\lambda}{N.A.} \quad (2)$$

With the measurement parameters used in our experiment, i.e. λ = 632.8 nm and *N.A.* = 0.9, Eqs. (1) and (2) yield d_{resol} = 429 nm and r_{focus} = 352 nm.

A practical way to estimate the focus diameter in a scanning microscopy experiment is the evaluation of an intensity profile obtained by scanning across a microscopic structure that is considered to be much smaller than the focus spot. If this is fulfilled, the microscopic structure acts as a probe, which is scanned through the light focus resulting in the intensity distribution inside the focus spot. This method was applied to the evaluation of a Raman map of $CuInSe_2$ (**Fig. S2**). In this particular mapping experiment, a pixel size of only 50 nm × 50 nm was used. All other relevant measurement parameters were typical values also applied in other mapping experiments described here (632.8 nm,

1 mW laser power, 100×/N.A. = 0.9 objective, 5 s per pixel, 200 × 200 pixels). **Fig. S2 c** reveals the line profile across a sharp feature in the intensity map of the 174-cm^{-1} band. A Gauss fit of the profile has a full width at half maximum (FWHM) of approx. 340 nm.

The lateral resolution can also be estimated by evaluating the change in signal intensity during a scan across a sharp edge in a sample. The resolution is directly connected to the distance over which the signal intensity increases from 10 to 90% of the final intensity, which simply corresponds to the full width at half maximum (FWHM) of the line-spread function (first derivative of the edge response). This method was recently described for tip-enhanced Raman imaging[19] and was applied here to the $CuInSe_2$ map shown in **Fig. S2 c**. The sigmoidal function fitted to the data (**Fig. S2 d**) yields a distance of approx. 410 nm between the 10% and 90% intensity values.

Summarizing these results we can experimentally confirm the theoretical values and the lateral resolution of this Raman mapping experiment is on the order of 400 nm.

<u>Depth resolution of Raman microspectroscopy</u>

When discussing the depth resolution of Raman microspectroscopy we have to distinguish between opaque and transparent samples. In opaque samples the sample volume contributing to the Raman signal is limited by the optical penetration depth and the lateral resolution. With an absorption coefficient on the order of 150 000 cm^{-1} in the spectral range around 600 nm[24] we get an optical penetration depth of approx. 70 nm for $CuInSe_2$, describing the distance over which the incoming laser beam is attenuated to $1/e \approx 37\%$ of its initial intensity. Based on this, we estimate that only the top 100-nm layer of a $CuInSe_2$ sample contributes to the collected Raman signals.

In transparent samples, the sample volume contributing to the Raman signal is again laterally defined by the lateral resolution. The depth resolution – at first glance defined by the focus depth – can be improved by the use of a confocal pinhole and is known to be on the order of micrometers[25]. Having a sample with an optical penetration depth far below the depth resolution offers the possibility to – very similar to the determination of the lateral resolution described above – scan the sample through the laser focus and map the signal contributions at different depths. This experiment was performed with different apertures of the confocal pinhole. The Raman signal intensities plotted as functions of depth are given in **Fig. S3**. The widths of the resulting Gauss-like profiles give an estimate of the depth resolution of the method if applied to transparent samples, reaching approx. 5 μm with the smallest apertures of 20-50 μm.

<u>Non-destructive character and reproducibility of the Raman measurements</u>

Focusing visible laser light onto a highly absorbing material always implies the risk of changing the internal structure or even damaging the material due to local photothermal heating. In the case of $CuInSe_2$ (632.8 nm laser wavelength and 100×/N.A. = 0.9 objective lens) we observed visible sample damages within a few seconds of laser exposure with powers of > 4 mW in the form of darkened spots of

approximately the size of the focus spot (**Fig. S1 a**) and at 10 mW in the form of "craters" with diameters of up to a few microns (**Fig. S1 b**). At 1 mW no visible sample damage was observed, even after several hours of exposure.

We consider the Raman spectra a much more sensitive indicator for alterations of the internal structure of a material, because changes in the Raman spectra might appear long before damages become visible on the sample surface. **Fig. S4 a** shows 10 spectra of $CuInSe_2$ that were consecutively measured on the same sample spot. At 1 mW laser power, no change in the spectra was observed proofing the non-invasive/non-destructive character of this method.

Further to the reproducibility of single-spot measurements, the reproducibility of whole images is another important criterion to proof the concept of a scanning microscopy experiment. As revealed in **Fig. S5**, Raman maps were collected on overlapping areas of a $CuInSe_2$ surface. Three small maps (**Figs. S5 c-e**) consisting of 40 × 40 pixels were collected first, followed by the 125 × 125-pixel, 25 × 25-μm^2 map shown in **Fig. S5 b**. Not only the microstructure but also the Raman intensities, i.e. the colors in the maps, are reproduced proofing both, the reproducibility and non-invasiveness of the method.

Potential to improve measurement speed

The limiting factor for the measurement speed in this experiment is the acquisition time needed to collect a single Raman spectrum. Typical acquisition times per pixel (i.e. per spectrum) in this study ranged from 3 s to 5 s, leading to collection times for Raman maps of, e.g., 80 min for 40 × 40 pixels (**Fig. S4 c-e**), approx. 23 h for 125 × 125 pixels (**Fig. S4 b**), up to 56 h for 200 × 200 pixels (**Fig. 2** and **Fig. S2**). This underlines the importance of improving measurement speed for the practical use of this method in the future. Indeed, Raman scattering cross sections vary a lot for different materials and therefore significantly shorter (but potentially also longer) acquisition times might be intrinsic to the analysis of materials other than $CuInSe_2$. Also, the absorbances (causing photothermal heating) and damage thresholds of other materials are different, potentially allowing the use of higher laser powers. One might also optimize the acquisition time of $CuInSe_2$ spectra to find the lowest signal-to-noise ratio necessary to detect the two bands evaluated here.

Further to these obvious options for improvement, there are two measurement parameters, which do not lower the signal intensity and have the potential to – if optimized together – decrease the mapping duration by an order of magnitude. First, we should point out that with pixel sizes of typically 200 nm × 200 nm we purposely oversampled the optical resolution, which was shown to be on the order of 400 nm. Using double the pixel size in both lateral directions would reduce the time of a mapping experiment performed on the same sample area by a factor of 4. Second, with a spectrometer grating with 1800 grooves mm^{-1} we used the highest spectral resolution accessible with our instrument in order to achieve the best possible separation of the Raman modes. Furthermore, the high spectral resolution is needed if local strain should be measured simultaneously to crystal orientation mappings as described below. Depending on the actual sample material a lower spectral resolution might be sufficient

to separate the evaluated Raman bands. As with higher spectral resolution, the Raman scattered light of a single band is distributed over more detectors (i.e., CCD pixels), the intensity necessarily decreases. On the other hand, with a reduction of the spectral resolution, spectra with the same intensity can be collected with shorter acquisition time. This is shown in **Fig. S4**. The use of the 300-mm^{-1} grating instead of the 1800-mm^{-1} grating yields spectra with the approx. same intensity within only one fifth of the time. In the present case of $CuInSe_2$, even with lower spectral resolution the two most prominent Raman bands evaluated in this study can still be separated, but baseline separation cannot be achieved with this grating.

Fig. S6 shows the influence of changes of spectral resolution and spatial resolution on the resulting Raman maps, all of them collected on the approximately same area of a $CuInSe_2$ sample. In **Fig. S6 a**, typical parameters of 250 nm × 250 nm pixel size, 1800-mm^{-1} grating and 5 s acquisition time per pixel were applied. The two maps revealing the distributions of the 174-cm^{-1} (red) and 214-cm^{-1} band intensities show a large grain in the center of this area with grain boundaries in the top right and bottom left corners of the image. Collection of the whole map took 5 h. Reduction of the spectral resolution by using the 300-mm^{-1} grating (shown in **Fig. S6 b**) allowed the reduction of the acquisition duration to 1 s per pixel while keeping the obtained Raman intensities approximately constant. The Raman images reveal the almost same structural features. It should be taken into account that the intrinsic drift of the sample stage caused the structures to move towards bottom left as compared to **Fig. S6 a**. Only the partial spectral overlap of the two bands plotted here caused the relative intensities to change in the maps. Thus, in **Fig. S6 b**, the central part of the green 214 cm^{-1} map appears brighter than in **Fig. S6 a**. Additional reduction of spatial resolution by increasing the pixel size from 250 × 250 nm^2 to 500 × 500 nm^2 gained an additional factor of four in measurement speed, and the collection time of the map shown in **Fig. S6 c** was only 15 min. Indeed, a significant reduction of image quality is the consequence, but still, the grain boundary in the top right corner is clearly visible.

Based on this experiment, we propose to use baseline-separated bands, if quantitative data regarding crystal orientations should be obtained. If the individual crystals in a polycrystalline material should only be visualized without extraction of quantitative data, reduced spectral resolution might be enough, which in our case improved measurement speed by a factor of five. If only large crystals are studied or if limited image quality is enough, measurement speed can be further improved by increasing the pixel sizes. Note that in other materials, the main Raman bands that are sensitive to crystal orientations might intrinsically appear better separated in the spectra, and a lower-resolution grating might be used without any compromises to obtain baseline-separated peaks (see, for example, our previous qualitative study of crystal orientations of β-Ca_2SiO_4 in Ref. 14).

As shown, both options together, i.e. increase of the pixel size and reduction of the spectral resolution, can potentially reduce the duration of mapping experiments by a factor of 20. Therefore, collection of 40 × 40-pixel maps within less than 5 min, of 125 × 125-pixel maps within approx. 1 h and even of 200 × 200-pixel maps within 2.5 h comes into reach.

Contributions of B_2 and E modes to the peak at 214 cm^{-1}

In addition to the theoretical Raman intensities at 214 cm^{-1} considering only contributions from the B_2 mode (**Figs. 4 a-e**), the simulated Raman intensities are also given assuming contributions from only the E mode as well as from a linear combination with equally weighted contributions from B_2 and E modes in **Fig.S7 a-e**. Again, the theoretical intensities were normalized to the maximum value, for the two polarization directions indicated in **Figs. 3 a-d**. It is apparent that the experimental Raman intensities for all five grains (**a-e**) can not be described well assuming contributions from only the B_2 mode, from only the E mode, or from a linear combination of these two modes. Therefore, an unambiguous assignment of the Raman intensities at 214 cm^{-1} to one specific mode valid for all grains in the specimen is not possible.

Strain measurements

Raman microspectroscopy has also the potential to determine the distribution of strain in a sample and even within individual grains. Local strain leads to small shifts of Raman band positions of typically < 1 cm^{-1}. Strain measurements by Raman spectroscopy are commonly used for silicon and even strain distribution mapping of this material has recently been demonstrated.[12] The wavenumber resolution – corresponding to the distance of two CCD pixels expressed in wavenumbers – of our instrument (with the 1800-mm^{-1} grating at 632.8 nm) is about 0.3 cm^{-1}, which is equivalent to a strain value in the order of 10^{-3}. However, the authors of Ref. 12 reported that fitting procedures can improve the wavenumber resolution down to approx. 0.05 cm^{-1}. This opens up the possibility to conduct strain measurements simultaneously to orientation distribution measurements even for strain values of below 10^{-3}.

Theoretical model

Group theory analysis of the lattice vibrations in $CuInSe_2$ (D_{2d} space group) at the Γ point has been performed[17, 26]. The following symmetry representation for the optical modes is obtained:

$$\Gamma = A_1 \oplus 2A_2 \oplus 3B_1 \oplus 3B_2 \oplus 6E. \qquad (3)$$

All these modes are Raman active, except silent A_2 modes. $3B_2 \oplus 6E$ modes are IR active and possess LO-TO splitting. E modes are double degenerated due to the equivalence of [100] and [010] directions.

For a given polarization geometry and crystal orientation, the intensity of a Raman mode, I, can be calculated from the Raman tensor, $\Re$, using the following equation[27]:

$$I \propto \left| v_i \Re' v_s \right|^2, \qquad (4)$$

where v_i and v_s are the unit polarization vectors for the incident and scattered light. Because we probe the Raman modes intensity for the specific (103) and (116) $CuInSe_2$ grains (i.e., grain 1 and grain 2) we transformed the Raman tensor for the laboratory frames via

$$\Re' = \Phi \Re \tilde{\Phi}, \tag{5}$$

where Φ and $\tilde{\Phi}$ are Euler's and inverse Euler's matrixes and $\Re$ is Raman tensor in crystal frame (XYZ)[28], where axes are [1 0 0], [0 1 0] and [0 0 1]. For clarity $\Re$ of the different modes of $CuInSe_2$ are listed in **Table S1**. In case of the laboratory frame for the (103) plane, we chose the laboratory axis X' along [4 0 3], the axis Y' along [0 1 0], and the axis Z' along [-3 0 1]. The Euler matrix is given by

$$\Phi_{103} = \begin{pmatrix} \cos\varphi_1 & 0 & -\sin\varphi_1 \\ 0 & 1 & 0 \\ \sin\varphi_1 & 0 & \cos\varphi_1 \end{pmatrix} \tag{6}$$

, where $\varphi_1 = a\cos(2/\sqrt{13}) \approx 56^\circ$ is an angle on which we rotate clockwise the crystal frame (XYZ) along Y to obtain the laboratory frame (X'Y'Z') associated with the (103) plane.

For the case of the (116) plane the Euler matrix is

$$\Phi_{116} = \begin{pmatrix} \cos\varphi_2\cos\theta & -\cos\varphi_2\sin\theta & -\sin\varphi_2 \\ \sin\theta & \cos\theta & 0 \\ \sin\varphi_2\cos\theta & -\sin\varphi_2\sin\theta & \cos\varphi_2 \end{pmatrix} \tag{7}$$

, where $\varphi_2 = a\cos(\sqrt{2/11}) \approx 65^\circ$ and $\theta = \pi/4$. The laboratory frame (X''Y''Z'') for the (116) plane can be obtained from the crystal frame (XYZ) by a two-step rotation transformation. The first step is a counter-clockwise rotation of the (XYZ) frame about Z through θ, and the second step is a clockwise rotation about Y'' through φ_2. After this transformation, X'' is [2 2 3], Y'' is [-1 1 0] and Z'' is [-3 -3 1]. The transformations for (1 1 12), (1 6 12), and (1 4 10) planes are similar to the considered (1 1 6)-plane so that we omitted intermediate steps. All rotation angles and the chosen laboratory frames were summarized in **Table S2.**

Finally, by combining Eqs. 5 and 6, we can derive formulae for the in-plane dependency of the Raman intensities from Eq. (4). In the present work, we performed Raman scattering measurements in parallel configuration, i.e., v_i and v_s vectors are

$$v_i = \begin{pmatrix} 0 & \sin\beta & \cos\beta \end{pmatrix} \qquad v_s^{\|} = \begin{pmatrix} 0 \\ \sin\beta \\ \cos\beta \end{pmatrix}, \tag{8}$$

where β is the angle between the Y' axis or Y'' axis and the direction of the light polarization. The calculated intensities for all Raman modes in parallel geometry are listed in **Table S3**. Note that intensities for E-type modes were calculated as a sum of E_x and E_y-type symmetry mode intensities with the weight parameter equal to 0.5 (Ref. 28). Since the calculated in-plane intensities for the B_1, B_2 and E-type modes are directly proportional to the corresponding tensor elements *c*, *d* and *e*, respectively, the line shape of these mode intensities is only depended on β. In contrast, for the A_1 mode in-plane intensity the ratio of the *a* and *b* tensor elements and β determine the line shape. It is interesting to note that in case of $a \approx b$ the intensity of A_1 modes would be constant at any value of β for all the considered grains here (see **Table S3**).

Table S3 summarizes normal and in-plane directions for grains 1 to 5 (**Fig. 3**), which include the polarization directions of the laser employed for Raman mapping experiments (see **Fig. 3**). The β angles given there and the corresponding intensity values extracted from the Raman maps as described above were plotted as circles in **Fig. 4** for comparison with theoretical trends.

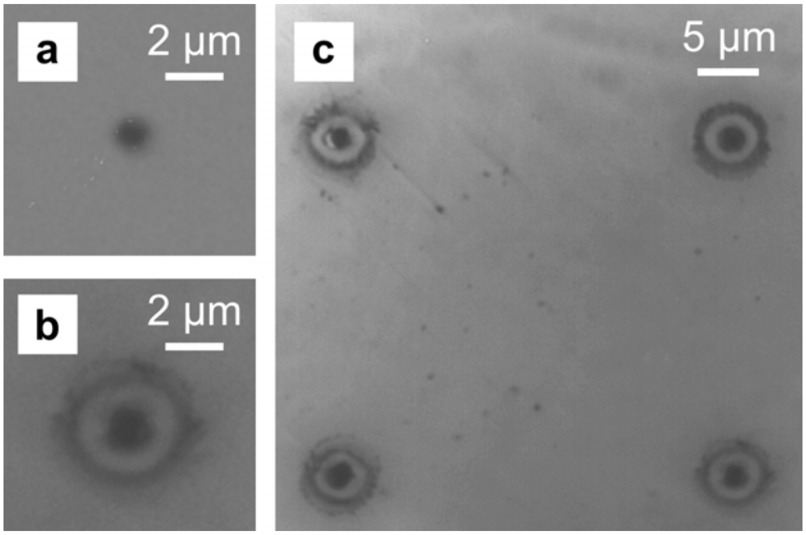

Fig. S1.

a: Damaged spot on a $CuInSe_2$ sample after 10 s laser irradiation with 5 mW power. **b:** Another sample spot after 10-mW laser irradiation for 10 s. **c:** Purposely damaged spots on a $CuInSe_2$ sample for marking an area that has been investigated by Raman microspectroscopy for further analysis by EBSD. The marks are placed outside the corners of the investigated area and were generated under the same conditions as spot **b**. Actually, **b** is a magnified version of the bottom right corner of **c**.

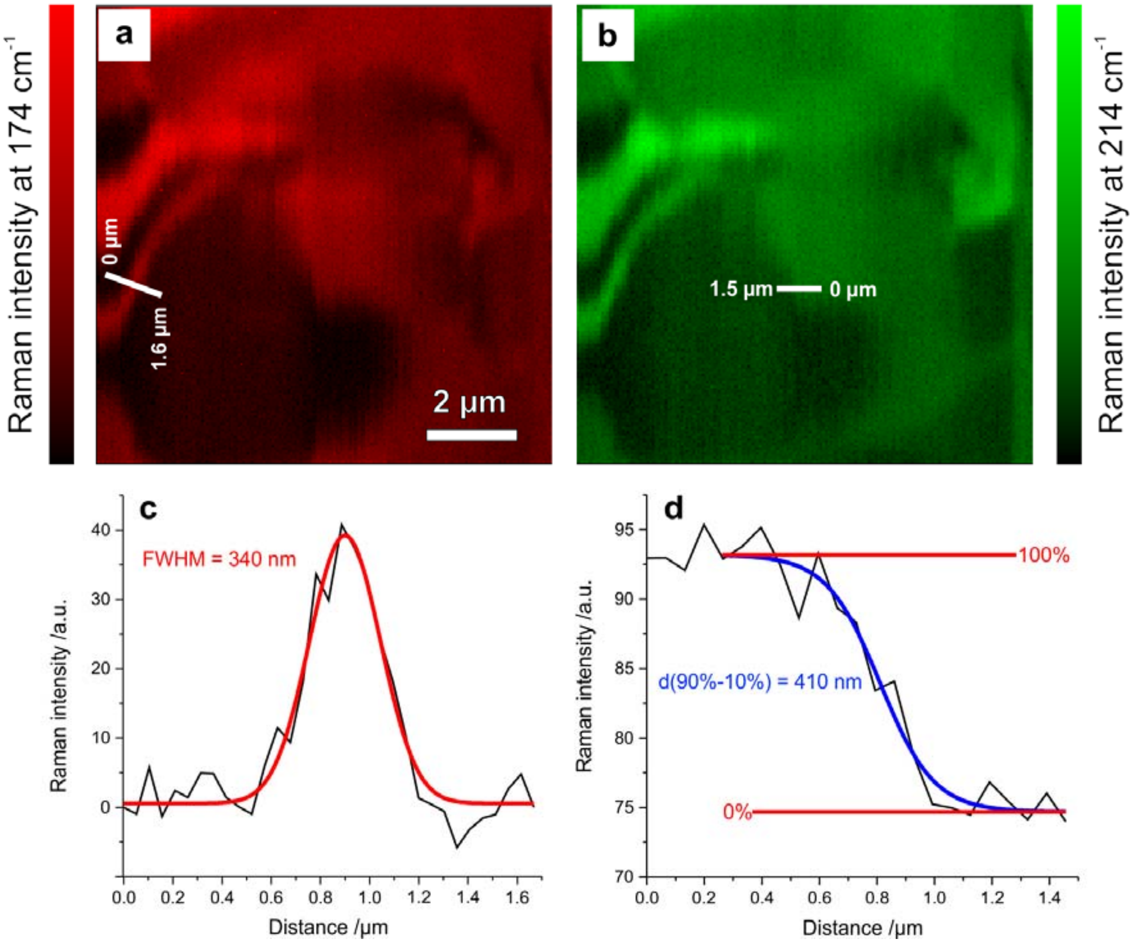

Fig. S2.

a, b: High-resolution Raman maps of $CuInSe_2$ sample consisting of 200 × 200 pixels on an area of 10 × 10 μm^2 (50 nm step size) revealing the distributions of the Raman intensities at 174 cm^{-1} and 214 cm^{-1}. White lines mark the places where the intensity profiles shown in **c** and **d** were derived from maps **a** and **b**. **c:** The lateral resolution of the Raman mapping experiment has been estimated based on the full width at half maximum (FWHM) of a Gauss fit through the intensity profile taken at the thinnest feature of the map. **d:** Alternatively, the lateral resolution can be determined by fitting a sigmoidal function to an edge response and calculating the lateral distance between 90% and 10% values.

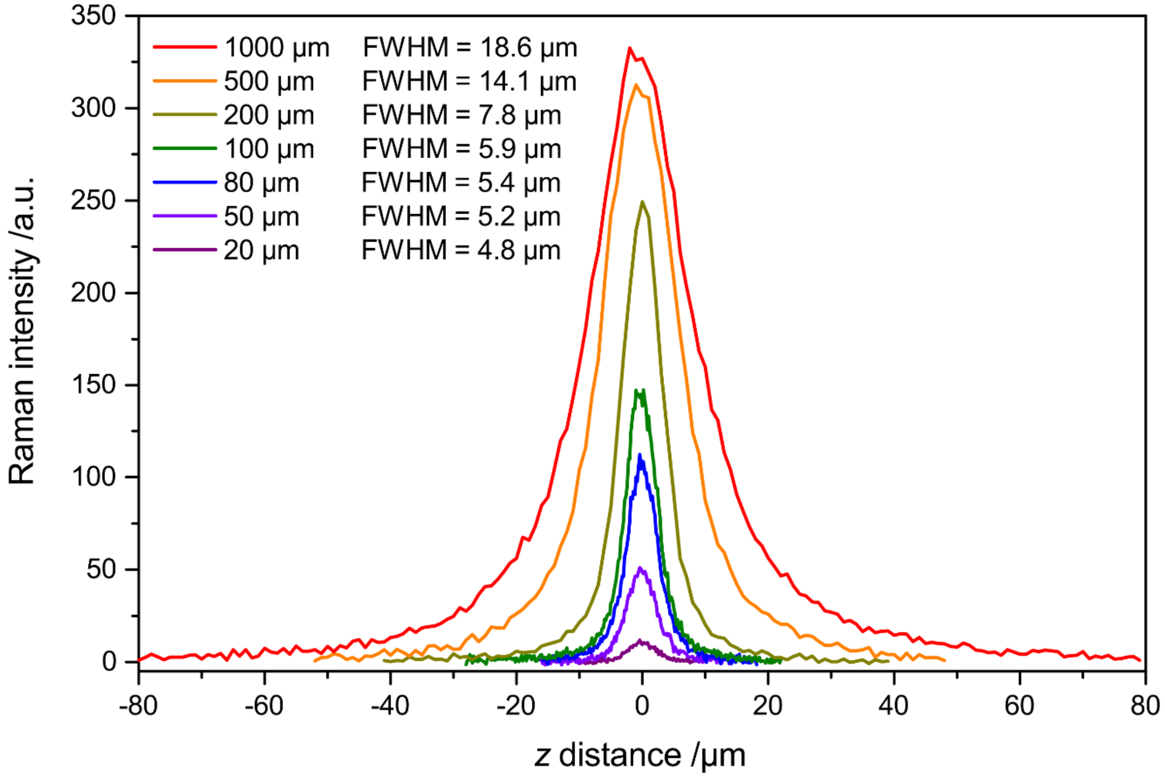

Fig. S3.

Signal intensities of the A_1 mode of $CuInSe_2$ collected in line scans along the depth axis (i.e., the *z* axis) with different pinhole apertures ranging from 20 µm to 1000 µm (full opening). As $CuInSe_2$ is opaque at 632.8 nm with a penetration depth on the order of only 100 nm, this measurement yields a good estimation of the depth resolution of confocal Raman microspectroscopy if the method would be applied to transparent samples (FWHM values were derived from Gauss fits). All data was acquired with the typical measurement parameters applied in this study, i.e. 632.8 nm laser wavelength and 1 mW power, 100 ×/N.A. =0.9 objective lens, and 5 s acquisition time per spectrum.

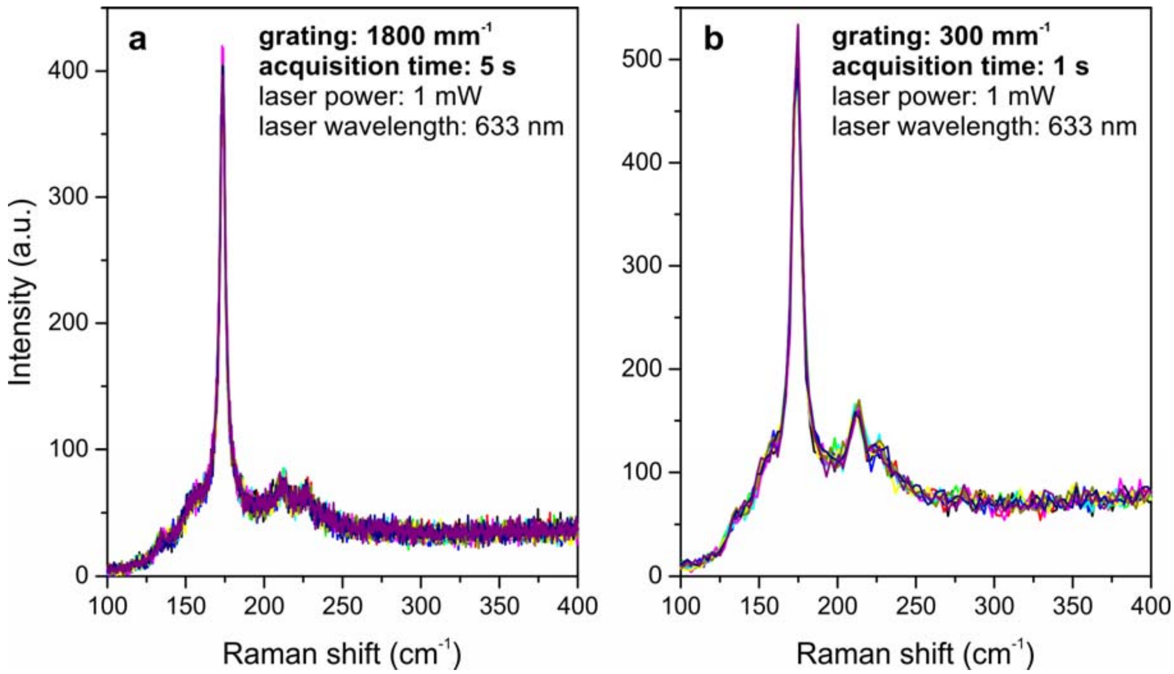

Fig. S4.

a: 10 repeated Raman measurements performed on the same spot of a $CuInSe_2$ sample with a 1800-mm^{-1} grating and 5 s acquisition time. **b:** The same experiment was repeated with a 300-mm^{-1} grating and 1 s acquisition time. The repetitions demonstrate the non-destructive/non-invasive character of the measurements performed with 1 mW laser power at 632.8 nm wavelength.

The comparison of **a** and **b** reveals the possibility to reduce the measurement time by a factor of 5 by reducing the spectral resolution. With the 300-mm^{-1} grating, the spectral resolution was approx. 2.5 cm^{-1}/CCD pixel at the position of the A_1 mode, which is still enough the separate the two most prominent bands in the spectrum. The spectral resolution of approx. 0.3 cm^{-1}/CCD pixel offered by the 1800-mm^{-1} grating (see **a**) yields spectra of higher quality allowing, for example, to additionally detect small band shifts appearing due to local strain, but 5 times the acquisition time is needed to achieve the approximately same signal intensities as in case **b**.

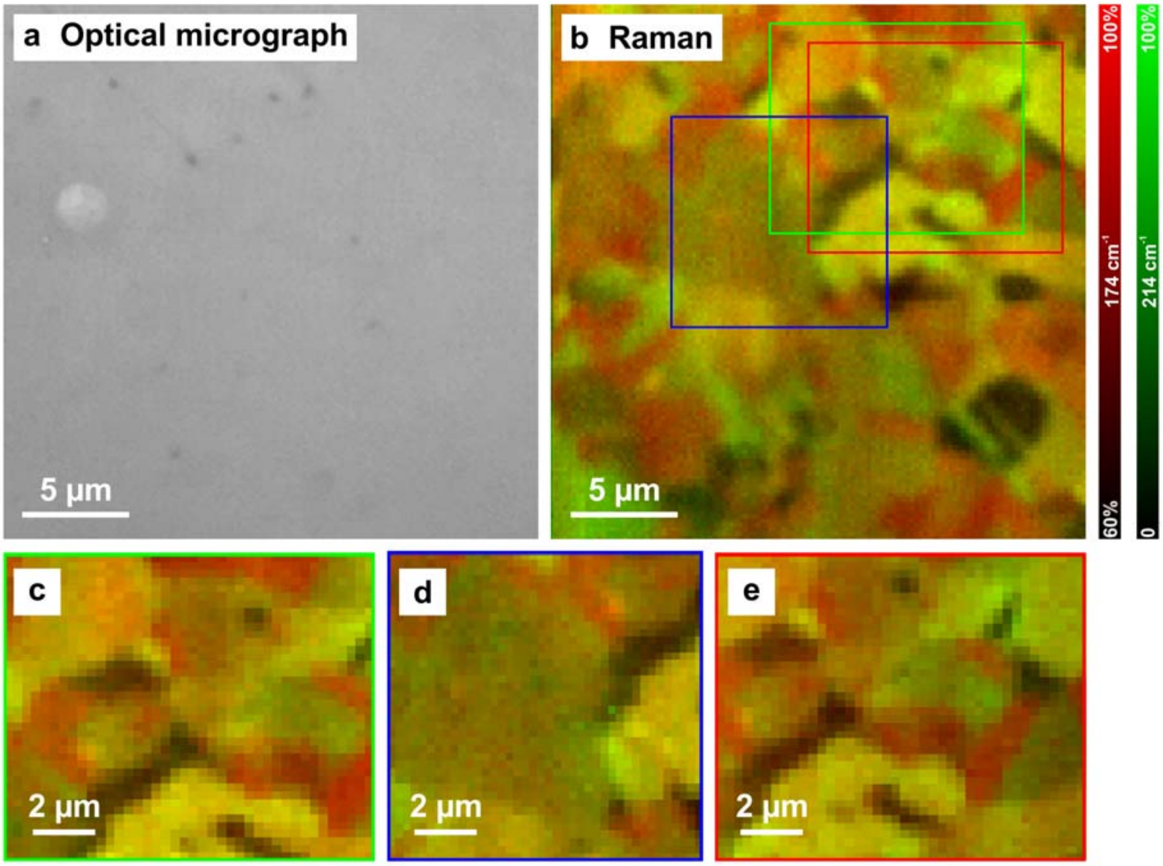

Fig. S5.

Reproducibility of the Raman mapping experiments. **a:** Optical micrograph of the investigated area of a $CuInSe_2$ sample. **b:** Raman map collected on the same area. **c-e:** Raman maps collected on smaller, overlapping areas. The Raman measurements were performed in the order **e**-**c**-**d**-**b**. With an acquisition time of 3 s/pixel (1800-mm^{-1} grating, 1 mW, 632.8 nm), collection of maps **c**-**e** took 80 min each, and map **b** took more than 23 h. Not only the same microstructures are repeatedly obtained, also the colors are reproduced demonstrating the reproducibility of Raman intensities, as also shown in **Fig. S4** for individual spectra.

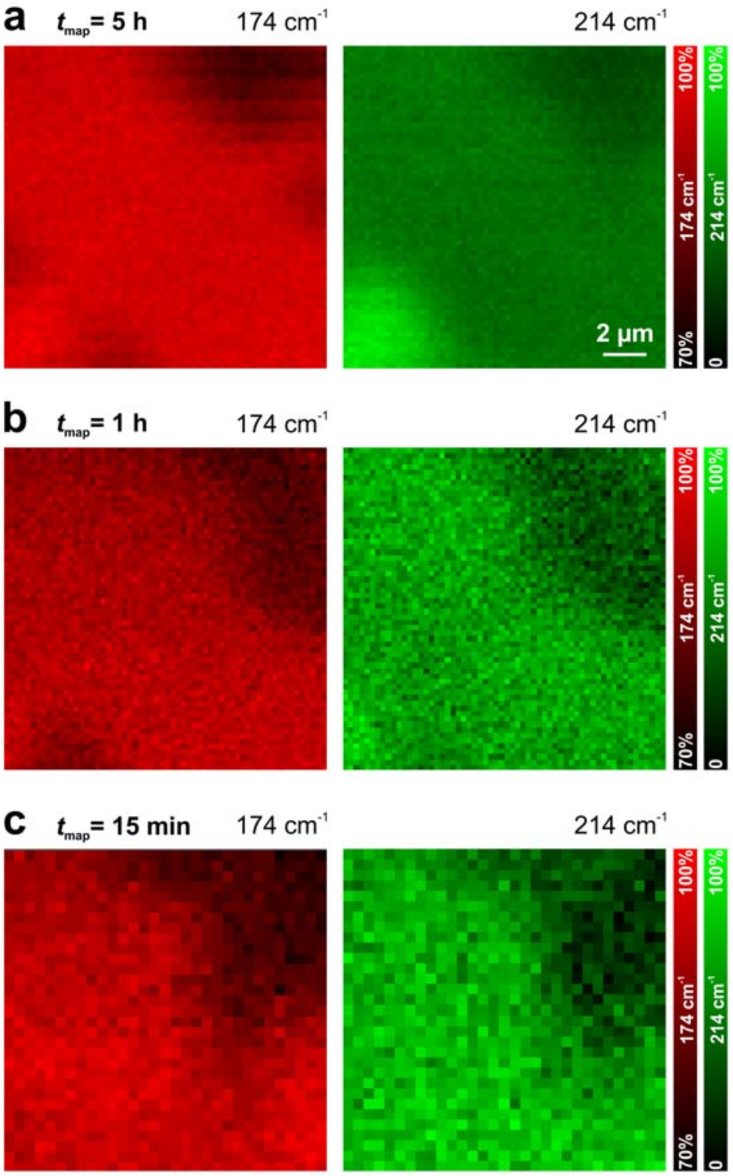

Fig. S6.

Three Raman maps collected on the almost same area of a $CuInSe_2$ sample showing the influence of some measurement parameters and the potential to reduce experiment time.

a: Typical measurement parameters: λ = 632.8 nm, 250 nm × 250 nm pixel size, grating with 1800 lines mm^{-1}, 5 s acquisition time per pixel, resulting in a collection time for the whole 60 × 60-pixel map of 5 h.

b: Reduction of spectral resolution by using a 300-mm^{-1} grating allowed to decrease the acquisition time per pixel to 1 s, while maintaining the almost same level of Raman intensities. Collection of the 60 × 60-pixel map took 1 h.

c: Reduction of both, spectral and spatial resolution. By applying 500 nm × 500 nm-sized pixels to the same area, the pixel number was decreased to 30 × 30, and the mapping experiment took only 15 min with 1 s acquisition time per pixel.

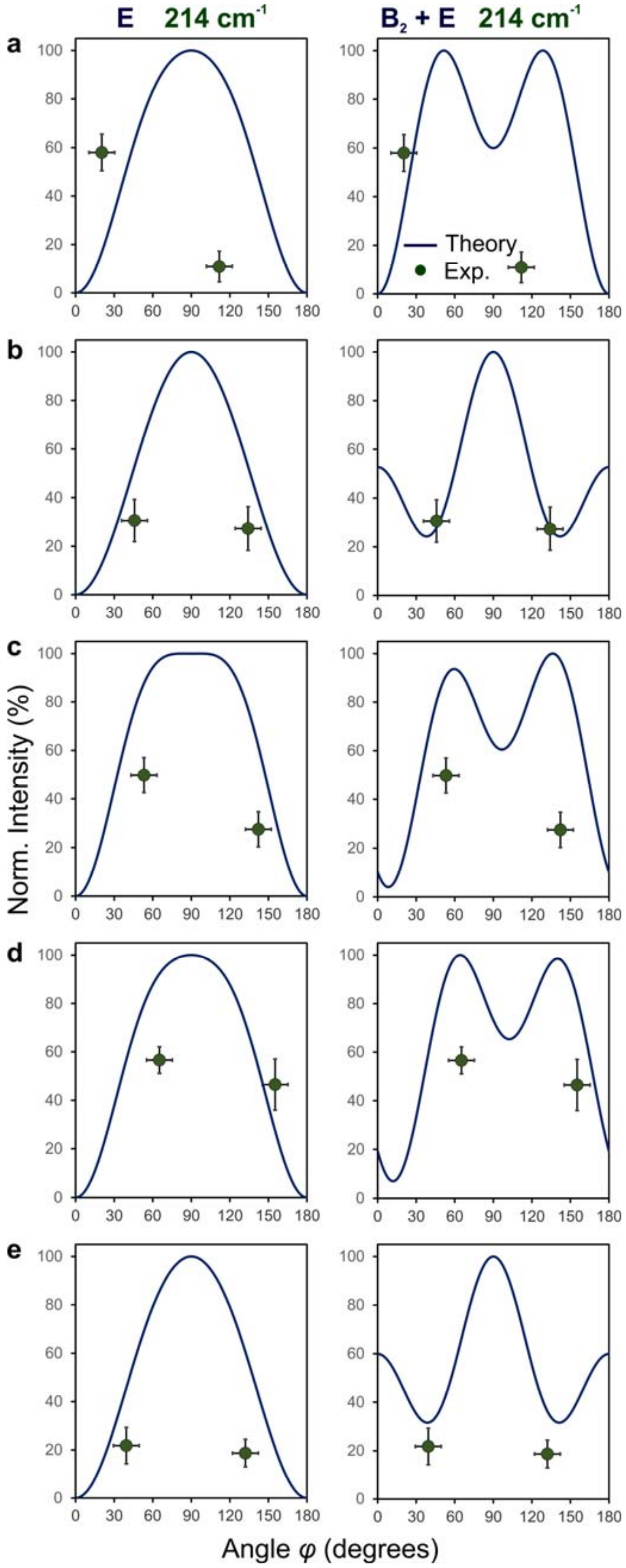

Fig. S7.

Theoretical Raman intensities at 214 cm^{-1}, normalized to the maximum value, for the two polarization directions indicated in **Figs. 3 a-d**, divided into contributions from the E mode (left column) as well as from a linear combination with equally weighted contributions from B_2 and E modes (right column). The Raman intensities differ depending on the orientation of the $CuInSe_2$ crystal lattice. The measured Raman intensities for the crystal orientations of grains 1-5 (**a-e**) are represented by green circles (214 cm^{-1}).

Table S1.

Raman tensors for A_1, B_1, $B_2(z)$, E(x) and E(y) symmetry modes in $CuInSe_2$.

A_1	B_1	$B_2(z)$	E(x)	E(y)
$\begin{pmatrix} a & 0 & 0 \\ 0 & a & 0 \\ 0 & 0 & b \end{pmatrix}$	$\begin{pmatrix} c & 0 & 0 \\ 0 & -c & 0 \\ 0 & 0 & 0 \end{pmatrix}$	$\begin{pmatrix} 0 & d & 0 \\ d & 0 & 0 \\ 0 & 0 & 0 \end{pmatrix}$	$\begin{pmatrix} 0 & 0 & 0 \\ 0 & 0 & e \\ 0 & e & 0 \end{pmatrix}$	$\begin{pmatrix} 0 & 0 & e \\ 0 & 0 & 0 \\ e & 0 & 0 \end{pmatrix}$

Table S2.

Crystallographic directions of the laboratory frame (X''Y''Z'') as well as the φ_2 and θ rotation angles selected for the (1 1 6) and (1 1 12); (1 6 12) and (1 4 10) -planes.

X''	Y''	Z''	φ_2	θ
**plane (1 1 6)**				
[2 2 3]	[-1 1 0]	[-3 -3 1]	$a\cos(\sqrt{2/11}) \approx 65°$	$\pi/4$
**plane (1 1 12)**				
[1 1 3]	[-1 1 0]	[-6 -6 1]	$a\cos(\sqrt{1/19}) \approx 77°$	$\pi/4$
**plane (1 6 12)**				
[1 6 3]	[-6 1 0]	[-12 -72 37]	$a\cos(\sqrt{37/73}) \approx \pi/4$	$a\cos(\sqrt{1/37}) \approx 81°$
**plane (1 4 10)**				
[2 8 5]	[-4 1 0]	[-10 -40 17]	$a\cos(\sqrt{17/42}) \approx 50°$	$a\cos(\sqrt{1/17}) \approx 76°$

Table S3.

Angular dependencies of the intensities of the Raman modes in parallel configuration.

A_1	B_1	B_2	E
**plane (1 0 3)**			
$\left[a\cos\beta^2 + \frac{1}{13}(9a + 4b)\sin\beta^2\right]^2$	$\left[-\frac{1}{13}c(2 + 11\cos(2\beta))\right]^2$	$\left[\frac{6d\cos\beta\sin\beta}{\sqrt{13}}\right]^2$	$\frac{1}{2}\left[\frac{4e\cos\beta\sin\beta}{\sqrt{13}}\right.$ [illegible] $+ \frac{1}{2}\left[\frac{12}{13}e\sin\beta^2\right.$ [illegible]
**plane (1 1 6)**			
$\left[\frac{1}{11}(10a + b + (a - b)\cos(2\beta))\right.$ [illegible]	$\left[\frac{3c\sin(2\beta)}{\sqrt{11}}\right]^2$	$\left[\frac{1}{11}d(1 + 10\cos(2\beta))\right]^2$	$\frac{1}{2}\left[\frac{2}{11}e(\sqrt{11}\cos\right.$ [illegible] $- 3\sin\beta)\sin\beta$ [illegible] $+$ $\frac{1}{2}\left[\frac{2}{11}e(\sqrt{11}\cos\right.$ [illegible] $+ 3\sin\beta)\sin\beta$ [illegible]
**plane (1 1 12)**			
$\left[\frac{1}{38}(37a + b + (a - b)\cos(2\beta))\right.$ [illegible]	$\left[\frac{3\sqrt{2}c\sin(2\beta)}{\sqrt{19}}\right]^2$	$\left[\frac{1}{38}d(1 + 37\cos(2\beta))\right]^2$	$\frac{1}{2}\left[\frac{1}{19}e(\sqrt{38}\cos\right.$ [illegible] $- 6\sin\beta)\sin\beta$ [illegible] $+$ $\frac{1}{2}\left[\frac{1}{19}e(\sqrt{38}\cos\right.$ [illegible] $+ 6\sin\beta)\sin\beta$ [illegible]

**plane (1 6 12)**			
$\left[a\cos(\beta)^2 + \frac{1}{73}(36a + 37b)\sin(\beta)\right.$	$\left[\frac{c(1295 + 3815\cos(2\beta) + 144\sqrt{73}\text{[illegible]}}{5402}\right.$	$\left[\frac{6d(37 + 109\cos(2\beta) - 35\sqrt{73}\text{[illegible]}}{2701}\right.$	$\frac{1}{2}\left[\frac{2}{73}e\left(\sqrt{73}\cos\text{[illegible]} - 36\sin\beta\right)\sin\text{[illegible]}\right.$ $+$ $\frac{1}{2}\left[\frac{12}{73}e\left(\sqrt{73}\cos\text{[illegible]} + \sin\beta\right)\sin\beta\right]^2$
**plane (1 4 10)**			
$\left[a\cos(\beta)^2 + \frac{1}{42}(25a + 17b)\sin(\beta)\right.$	$\left[\frac{5c(51 + 201\cos(2\beta) + 16\sqrt{42}\sin(\text{[illegible]}}{1428}\right.$	$\left[\frac{d(68 + 268\cos(2\beta) - 75\sqrt{42}\sin\text{[illegible]}}{714}\right.$	$\frac{1}{2}\left[\frac{1}{21}e\left(\sqrt{42}\cos\text{[illegible]} - 20\sin\beta\right)\sin\text{[illegible]}\right.$ $+$ $\frac{1}{2}\left[\frac{1}{21}e\left(4\sqrt{42}\cos\text{[illegible]} + 5\sin\beta\right)\sin\beta\text{[illegible]}\right.$

Table S4.
Normal and in-plane directions in grains 1-5 (see Fig. 3).

	grain 1 – (1 0 3) plane			grain 2 – (1 1 12) plane		
**normal direction**	[4 0 3]		X‘ axis	[1 1 3]		X‘‘ axis
**in-plane directions**	[0 1 0]	$\beta = 0°$	Y‘ axis	[-1 1 0]	$\beta = 0°$	Y‘‘ axis
	[-1 12 1]	$\beta = 20.4°$	polarization ⬌(*)	[0 -12 1]	$\beta = 134.2°$	polarization ⬍
	[-8 -4 3]	$\beta = 111.8°$	polarization ⬍	[12 0 -1]	$\beta = 45.8°$	polarization ⬌
	**grain 3 – (1 6 12) plane**			**grain 4 – (1 4 10) plane**		
**normal direction**	[1 6 3]		X‘‘ axis	[2 8 5]		X‘‘ axis
**in-plane directions**	[-6 1 0]	$\beta = 0°$	Y‘‘ axis	[-4 1 0]	$\beta = 0°$	Y‘‘ axis
	[12 8 -5]	$\beta = 53.2°$	polarization ⬌(*)	[-2 -2 1]	$\beta = 65.2°$	polarization ⬍
	[-10 8 -3]	$\beta = 142.2°$	polarization ⬍	[6 -4 1]	$\beta = 155.2°$	polarization ⬌
	**grain 5 – (116) plane**					
**normal direction**	[2 2 3]		X‘‘ axis			
**in-plane directions**	[-1 1 0]	$\beta = 0°$	Y‘‘ axis			
	[-1 -6 -1]	$\beta = 39.4°$	polarization ⬍			
	[-6 0 1]	$\beta = 132.1°$	polarization ⬌			

(*) polarization directions according to Fig. 3

References

1. Weber, W.H. & Merlin, R. (eds) *Raman Scattering in Materials Science*, in *Springer Series in Materials Science* (eds Hull, R. et al.), vol. 42 (Springer, 2000).

2. Bhagavantam, S. Effect of crystal orientation on the Raman spectrum of calcite. *Proc. Ind. Acad. Soc. A.* **11**, 62-71 (1940).

3. Loudon, R. The Raman effect in crystals. *Adv. Phys.* **13**, 423-482 (1964).

4. Hopkins, J.B. & Farrow, L.A. Raman microprobe determination of local crystal orientation. *J. Appl. Phys.* **59**, 1103-1110 (1986).

5. Mizoguchi, K. & Nakashima, S. Determination of crystallographic orientations in silicon films by Raman-microprobe polarization measurements. *J. Appl. Phys.* **65**, 2583-2590 (1989).

6. Yoshikawa, M., et al. Study of crystallographic orientations in the diamond film on cubic boton nitride using Raman microprobe. *Appl. Phys. Lett.* **57**, 428-430 (1990).

7. Kolb, G., Salbert, T. & Abstreiter, G. Raman-microprobe study of stress and local orientation in laser-crystallized silicon. *J. Appl. Phys.* **69**, 3387-3389 (1991).

8. Hayward, I.P., Baldwin, K.J., Hunter, D.M., Batchelder, D.N. & Pitt G.D. Direct imaging and confocal mapping of diamond films using luminescence and Raman scattering. *Diamond Rel. Mater.* **4**, 617-621 (1995).

9. Klose, M., et al. Raman scattering investigations of $CuInSe_2$ films deposited by coevaporation and laser ablation. *Proc. SPIE 2403*, *Laser-Induced Thin Film Processing* (ed Dubowski, J.J.), 239-250 (1995).

10. Habka, N., Barjon, J., Lazea, A. & Haenen, K. Stress in (110)-textured phosphorus-doped polycrystalline diamond studied by Raman and cathodoluminescence spectroscopies. *J. Appl. Phys.* **107**, 103531-1-4 (2010)

11. Kunz, T., Hessmann, M.T., Meidel, B. & Brabec, C.J. Micro-Raman mapping on layers for crystalline silicon thin-film solar cells. *J. Cryst. Growth* **314**, 53-57 (2010).

12. Becker, M., Sarau, G. & Christiansen, S. *Submicrometer-Scale Characterization of Solar Silicon by Raman Spectroscopy*, in *Mechanical Stress on the Nanoscale: Simulation, Material Systems and Characterization Techniques* (eds Hanbücken, M. et al.) Ch. 14, 299-332 (Wiley, 2011).

13. Jeong, A.R., et al. Local current transport and surface potential of photovoltaic $Cu(In,Ga)Se_2$ thin films probed by multi-scale imaging methods, *Adv. Nat. Sci.: Nanosc. Nanotechn.* **4**, 015007; DOI:10.1088/2043-6262/4/1/015007 (2013).

14. Schmid, T. & Dariz, P. Determination and imaging of binder remnants and aggregates in historic cement stone by Raman microscopy. *J. Raman Spectrosc.* **44**, 882-891 (2013).

15. Fan, H.M., et al.. Orientation-Dependent Raman Spectroscopy of Single Wurtzite CdS Nanowires, J. Phys. Chem. C 112, 1865-1870 (2008).

16. Knight, K.S. Crystal structures of $CuInSe_2$ and $CuInTe_2$. *Mater. Res. Bull.* **27**, 161-167 (1992).

17. Tanino, H., et al. Raman spectra of $CuInSe_2$. *Phys. Rev. B* **45**, 13323-13330 (1992).

18. Tanino, H., Fujikake, H., Maeda, T. & Nakanishi, H. Determination of the crystal orientation of $CuInSe_2$ by Raman spectroscopy, *J. Appl. Phys.* **74**, 2114-2116 (1993)

19. Schmid, T., Opilik, L., Blum, C. & Zenobi, R. Nanoscale chemical imaging using tip-enhanced Raman spectroscopy: A critical review. *Angew. Chem. Int. Ed.* **52**, 5940-5954 (2013).

20. Kaufmann, C.A., Neisser, A., Klenk, R. & Scheer, R. Transfer of $Cu(In,Ga)Se_2$ thin film solar cells to flexible substrates using an in situ process control. *Thin Solid Films* **480-481**, 515-519 (2005).

21. Contreras, M.A., et al. High efficiency $Cu(In,Ga)Se_2$-based solar cells: Processing of novel absorber structures, *Proc. 24th IEEE Photovoltaics Specialists Conference, Waikola, Hawaii*, 68-75 (1994).

22. Rayleigh, L. Investigations in Optics, with Special Reference to the Spectroscope. *Philos. Mag.* **8**, 261-274 (1879).

23. Abbe, E. Beitraege zur Theorie des Mikroskops und der mikroskopischen Wahrnehmung. *Arch. Mikrosk. Anat. Entwicklungsmech.* 9, 413-468 (1873).

24. Theodoropoulou, S., Papadimitriou, D., Anestou, K., Cobet, C. & Esser, N. Optical properties of $CuIn_{1-x}Ga_xSe_2$ quaternary alloys for solar-energy conversion. *Semicond. Sci. Technol.* **24**, 015014-1-8 (2009).

25. Opilik, L., Schmid, T. & Zenobi, R. Modern Raman imaging: vibrational spectroscopy on the micrometer and nanometer scales. Ann*u. Rev. Anal. Chem.* **6**, 379-98 (2013).

26. Holah, G.D., Schenk, A.A., Perkowitz, S. & Tomlinson, R.D. *Infrared reflectivity of p-type $CuInTe_2$. Phys. Rev. B* **23**, 6288-6293 (1981).

27. Loudon, R. Theory of the resonance Raman effect in crystals. *J. Phys.* **26**, 677-683 (1965).

28. Pezzotti, G., Sueoka, H., Porporati, A.A., Manghnani, M. & Zhu, W. Raman tensor elements for wurtzitic GaN and their application to assess crystallographic orientation at film/substrate interfaces. *J. Appl. Phys.* **110**, 013527-1-10 (2011)
